# Unraveling the microbial mysteries: gut microbiota’s role in ulcerative colitis

**DOI:** 10.3389/fnut.2025.1519974

**Published:** 2025-02-10

**Authors:** Keyu Ren, Chunming Yong, Yanchun Jin, Shanwei Rong, Kuijin Xue, Bin Cao, Hongyun Wei

**Affiliations:** ^1^Department of Gastroenterology, The Affiliated Hospital of Qingdao University, Qingdao, China; ^2^Department of Emergency, The Affiliated Hospital of Qingdao University, Qingdao, China

**Keywords:** ulcerative colitis, gut microbiota, extraintestinal homeostasis, biological immunotherapy, fecal microbiota transplantation

## Abstract

Ulcerative colitis (UC) is a chronic inflammatory bowel disease characterized by persistent inflammation of the colon. Recent research has highlighted the significant role of gut microbiota in the pathogenesis and treatment of UC. This review aims to provide a comprehensive overview of the current understanding of the relationship between gut microbiota and UC. We discuss the involvement of gut microbiota in the onset of UC, including the dysbiosis observed in patients and its potential mechanisms. Additionally, the role of extra-intestinal microbiota in UC pathogenesis is explored, which has been less studied but is gaining attention. The influence of gut microbiota on the efficacy of biological immunotherapy for UC is also examined, highlighting how microbial composition can influence treatment outcomes. Furthermore, we review microbiota transplantation, and their potential benefits in UC management. Finally, we consider the combined use of antibiotics and biological agents in UC treatment, discussing their synergistic effects and potential drawbacks. This review underscores the importance of gut microbiota in UC and suggests that targeting microbial communities could offer new avenues for effective treatment.

## Graphical abstract

Schematic diagram of this review. **(A)** Schematic diagram illustrating the impact of gut microbiota homeostasis and skin integrity on gut microbiota and barrier integrity. **(B)** Schematic diagram illustrating the impact of probiotic or prebiotic treatments on gut microbiota. **(C)** Mechanistic model of the role of gut microbiota and intestinal barrier integrity in the development of intestinal inflammation.

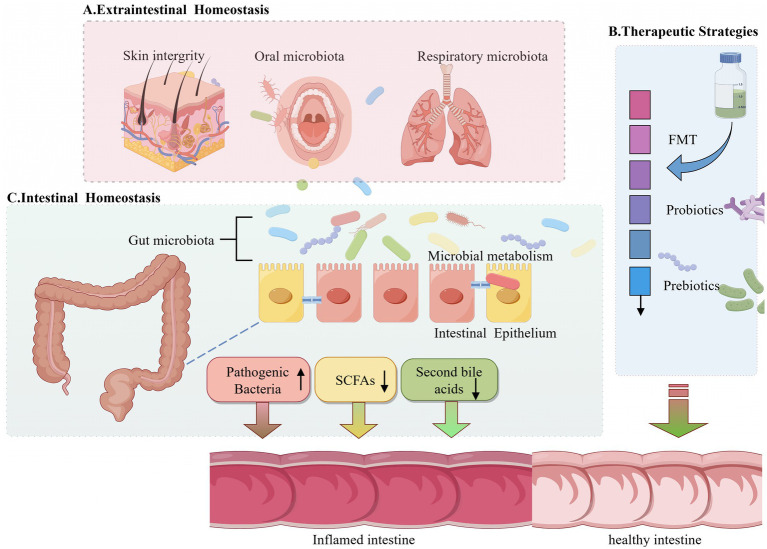

## 1 Introduction

The complex interplay between the gut microbiota and human health has attracted considerable scholarly interest in recent years. The gut microbiota, a diverse microbial community inhabiting the gastrointestinal tract, is integral to the maintenance of homeostasis and the modulation of various physiological processes. Dysbiosis, or alterations in the composition of the gut microbiota, has been implicated in the pathogenesis of numerous diseases, including inflammatory bowel diseases (IBD) such as ulcerative colitis (UC) (1).

Ulcerative colitis is a chronic inflammatory disorder marked by inflammation and ulceration of the colonic mucosa. The etiology of UC is multifactorial, encompassing genetic, environmental, and immunological components. Environmental factors are pivotal in determining the composition of the microbiome (2). Among these, diet stands out as a crucial environmental determinant capable of modifying the composition and metabolic activity of the gut microbiota, thereby influencing the incidence and progression of gastrointestinal diseases (3). Recent research has highlighted the critical role of gut microbiota in the pathogenesis and progression of UC. Dysbiosis in individuals with UC is characterized by a decrease in microbial diversity and an imbalance between commensal and pathogenic bacteria (4). This microbial dysregulation contributes to the compromise of the intestinal barrier, resulting in heightened intestinal permeability and subsequent inflammation.

Beyond the gut, the influence of microbiota extends to extraintestinal sites, affecting systemic immune responses and potentially contributing to the pathogenesis of extraintestinal manifestations of UC. For instance, the translocation of gut bacteria to secondary lymphoid organs and other tissues can modulate immune responses and exacerbate inflammation (5). This indicates the critical importance of comprehending the interactions between gut and extraintestinal microbiota within the framework of UC.

The influence of gut microbiota on the effectiveness of biological therapies for UC has been a focal point of extensive research. Biological therapies, including anti-TNF agents and immune checkpoint inhibitors, have significantly advanced the treatment paradigm for UC. Nevertheless, their therapeutic efficacy is frequently inconsistent, and recent studies indicate that the gut microbiota composition may affect treatment outcomes. Specific microbial signatures have been associated with better responses to biological therapies, underscoring the potential of microbiota-based biomarkers in predicting therapeutic efficacy (6).

Modulating the gut microbiota has emerged as a promising therapeutic strategy for UC. Approaches such as fecal microbiota transplantation (FMT), probiotics, and prebiotics aim to restore microbial balance and enhance intestinal health. Certain probiotics, including *Escherichia coli* Nissle 1917 and VSL#3, have been shown to successfully induce and sustain remission in UC (7, 8). VSL#3 has been shown to exert anti-inflammatory effects by enhancing the production of regulatory cytokines while diminishing the expression of pro-inflammatory cytokines and Toll-like receptors (TLRs) (9). Additionally, prebiotics have the potential to alter the composition of the intestinal flora, augment the population of beneficial bacteria, and decrease the pH level of the colon (10). FMT, in particular, has shown promise in inducing remission in UC patients by transferring healthy donor microbiota to the recipient, thereby re-establishing a healthy microbial ecosystem (11). Additionally, the use of antibiotics in combination with biological therapies has been explored to enhance therapeutic outcomes by targeting specific pathogenic bacteria and reducing inflammation (12).

In conclusion, the gut microbiota is integral to the pathogenesis and treatment of UC. Understanding the complex interactions between gut microbiota, host immune responses, and therapeutic interventions is crucial for developing effective strategies to manage UC. This review examines the existing knowledge regarding the role of both gut and extraintestinal microbiota in UC, the influence of microbiota on the efficacy of biological therapies, and the potential of microbiota modulation as a therapeutic strategy. Keywords like “microbiota or bacteria”, “ulcerative colitis”, “diet”, and “treatment” were used to search the Pubmed database. Through this exploration, our objective is to offer a comprehensive overview of the microbiota-UC axis and its implications for future research and clinical practice.

### 1.1 Gut microbiota and the occurrence of ulcerative colitis

The human gut hosts a complex microbial community that engages in diverse metabolic, physiological, and immunological functions. Dysbiosis as alterations in gut microbiome composition can be influenced by various factors, such as life style, hormonal changes, aging, antibiotic use, fasting/feeding status, and altered Paneth cells (13). Dysbiosis of gut microflora has been linked to the pathogenesis of intestinal disorders, including IBD and UC (14). The integrity of the intestinal mucosal barrier is contingent upon its interactions with gut microbiota. The commensal bacterial community plays a role in the pathogenesis of IBD, including UC, through mechanisms such as diminished biodiversity, abnormal gut microbiota composition, and altered spatial distribution (15). Dysbiosis leads to pore formation in the intestinal epithelium and activates pattern recognition receptors, thereby promoting local inflammation (16). This microbial imbalance can lead to increased gut permeability, allowing translocation of intestinal pathogens, antigens, and toxins into extra-intestinal sites, which further exacerbates inflammation (17).

Recent research has demonstrated that particular microbial species and their metabolites are integral to the development and progression of UC. For instance, the depletion of gut microbiota via broad-spectrum antibiotics can compromise gut barrier integrity and inhibit the antiviral immune response of T cells in the liver, highlighting the importance of gut microbiota in maintaining immune homeostasis (18). Additionally, research has substantiated the existence of bacteria in the gastrointestinal tract before birth and highlighted the significance of the lactation period in the development of gastrointestinal microbiota. This evidence suggests that the establishment of microbiota in early life is critical for physiological development and the maintenance of immunological homeostasis (19).

Moreover, gut microbiota dysbiosis is intricately linked to disturbances observed in UC. An increased abundance of Bacteroides 2 was observed in fecal water of patients with UC (20). In the faeces of UC patients in the mucosal healing state after infliximab treatment, the bacterial flora was characterized by increased *Faecalibacterium prausnitzii* and *Bacteroides fragilis* (21). In addition, natural products, such as indigo naturalis and bergenin, have been shown to alleviate UC by regulating gut microbiota. Indigo naturalis, a traditional Chinese medicine, alleviates dextran sulfate sodium (DSS)-induced colitis in rats by rectifying intestinal dysbiosis and enhancing fecal butyrate levels, which is positively associated with the relative abundances of Ruminococcus_1 and Butyricicoccus (12). Similarly, bergenin decreases the abundance of *Bacteroides vulgatus*, and ameliorates colitis symptoms in DSS-induced mice by inhibiting TLR4, modulating NF-κB and mTOR phosphorylation, and reducing branched-chain amino acid (BCAA) levels (22).

In conclusion, gut microbiota is crucial in the occurrence and progression of UC. Dysbiosis of gut microbiota leads to increased gut permeability, local and systemic inflammation, and impaired immune responses. Natural products and interventions targeting gut microbiota hold promise for the prevention and treatment of UC by reestablishing microbial equilibrium and enhancing gut barrier integrity.

### 1.2 Extraintestinal microbiota/extraintestinal organ integrity and the occurrence of ulcerative colitis

The role of extraintestinal microbiota in the pathogenesis of UC has garnered increasing attention in recent years. Extraintestinal microbiota, including those found in oral cavity, and respiratory tract, may influence gut health through various mechanisms. For instance, dysbiosis in microbiota can precipitate systemic inflammation, subsequently exacerbating intestinal inflammation. The oral microbiota has been implicated in the pathogenesis of UC (23). Oral pathogens can translocate to the gut, contributing to gut dysbiosis and inflammation (24). For example, *Klebsiella pneumoniae* and Klebsiella aeromobilis from the saliva of patients with UC could lead to severe intestinal inflammation in colitis-prone IL10−/− mice (25). The respiratory tract microbiota, although less studied, may also play a role in gut health (26). Inflammatory responses in the respiratory tract can result in systemic inflammation, which may exacerbate gut inflammation (27). Therefore, maintaining a balanced extraintestinal microbiota could be crucial for preventing and managing UC. In addition, the integrity of extraintestinal organ like skin is also important to maintain gut health. When germ-free Mice treated with DSS ware transplanted with fecal microbiota from mice with skin wounds, they showed higher intestinal inflammation compared to those received FMT from co-housed littermates without skin wounds (28). Gut microbiota in mice with skin injury was characterized with increased abundance of several bacterial species including Lachnospiraceae bacterium A4 and *Akkermansia muciniphila*, which is different from those without skin injury. In the imiquimod-induced psoriasis mouse model using Toll-like receptor (TLR) 7 ligand, there is damage to the integrity of the small intestine barrier and inflammatory changes in the small intestine (29). These studies indicated the relationship between skin integrity and gut health. A review summarized that the resident skin microbiota plays an important role in maintaining skin immune function by regulating interlukin (IL)-1 and T cells (30). These literatures indicate that skin symbiotic bacteria play an important role in regulating the integrity of the local skin barrier, which in turn affects the gut microbiome homeostasis. Disruption of the gut microbiome can lead to intestinal inflammation. Therefore, we hypothesized that the skin microbiome may indirectly influence the composition of the gut microbiome. However, whether dysbiosis existed in skin damage could cause intestinal inflammation is still need to be illustrated in the near future.

In conclusion, while the gut microbiota has been extensively studied in the context of UC, the role of extraintestinal microbiota is an emerging field of research. Understanding the complex interactions between different microbiota and their impact on gut health could lead to novel therapeutic strategies for UC. Future studies should focus on elucidating the mechanisms by which extraintestinal microbiota influence gut health and exploring potential interventions to modulate microbiota for the prevention and treatment of UC.

### 1.3 Influence of gut microbiota on the efficacy of biological immunotherapy for ulcerative colitis

The gut microbiota plays a crucial role in modulating the immune system and influencing the efficacy of biological immunotherapy in treating UC. Recent studies have underscored the substantial influence of gut microbiota composition on the therapeutic outcomes of immune checkpoint inhibitors (ICIs) and other immunotherapies. For instance, the presence of specific bacterial strains such as *Faecalibacterium prausnitzii* has been associated with improved responses to ICIs and reduced colitis severity in cancer patients undergoing immunotherapy (31). This bacterium is known for its anti-inflammatory properties and capacity to modulate the gut immune environment, thereby enhancing the efficacy of immunotherapy while mitigating adverse effects.

Moreover, the gut microbiota’s role in immune regulation extends to the modulation of immune-related adverse events (irAEs), which are common complications of biological therapies. Studies have shown that a diverse and balanced gut microbiota can reduce the incidence and severity of irAEs, including colitis, by maintaining intestinal homeostasis and promoting regulatory immune responses (32). For example, supplementation with specific probiotics or FMT has demonstrated effectiveness in the treatment of refractory ICI-related colitis, suggesting that microbiota modulation could be a viable strategy to enhance the safety and efficacy of biological immunotherapies (33).

Furthermore, the interaction between gut microbiota and immunotherapy is not limited to bacterial composition alone but also involves microbial metabolites. Short-chain fatty acids (SCFAs), such as butyrate, produced by gut bacteria have been shown to exert protective effects on the intestinal mucosa and enhance the anti-tumor immune response (34). A high-fiber diet can increase the abundance of beneficial bacteria such as Bacteroidetes (Porphyromonadaceae and Rikenellaceae) and Firmicutes (Lachnospiraceae), and promote the synthesis of SCFAs, thereby alleviating UC symptoms (35). In addition, these metabolites can modulate the function of immune cells, such as T cells and macrophages, thereby influencing the overall therapeutic outcome. For instance, butyrate has been found to improve the fitness and expansion of T cells ex vivo, which is critical for the success of adoptive cell therapies like CAR-T cell therapy (36).

In addition to SCFAs, other microbial-derived metabolites, such as secondary bile acids, have been implicated in modulating immune responses and influencing the efficacy of immunotherapy. Research has shown that alterations in bile acid metabolism, mediated by gut microbiota, can predict responses to anti-α4β7-integrin therapy in Crohn’s disease, a condition closely related to UC (37). These findings suggest that targeting microbial metabolism could be a novel approach to enhance the efficacy of biological therapies in UC.

Overall, the intricate relationship between gut microbiota and the immune system emphasizes the potential of microbiota-targeted interventions to improve the outcomes of biological immunotherapy in UC. By modulating the gut microbiota composition and its metabolic activities, it is possible to enhance therapeutic efficacy, reduce adverse effects, and ultimately achieve better clinical outcomes for patients with UC. Further research is needed to fully elucidate the mechanisms underlying these interactions and to develop effective microbiota-based therapeutic strategies.

### 1.4 Modulation of gut microbiota in the treatment of ulcerative colitis

The modulation of gut microbiota has emerged as a promising therapeutic strategy for the treatment of UC. Numerous studies have demonstrated that altering the composition and function of gut microbiota can significantly alleviate the symptoms of UC and promote intestinal health. For instance, ginsenoside compound K (GC-K) has been shown to alleviate dextran sulfate sodium (DSS)-induced colitis by increasing the abundances of Verrucomicrobia and Patescibacteria and decreasing Proteobacteria (38). In addition, treatment with GC-K was associated with a reduction in disease activity index (DAI) scores, a decrease in spleen weight, and an increase in colon length. The treatment also enhanced the expression of tight junction proteins and reduced pro-inflammatory cytokines, including TNF-*α*, IL-6, IL-1β, and IL-17. Furthermore, FMT experiments confirmed that GC-K-driven gut microbiota significantly relieved DSS-induced mice colitis model (38).

Another study highlighted the protective properties of turmeric polysaccharides (TPS) in mitigating DSS-induced UC. TPS administration improved pathological phenotypes, gut barrier disruption, and colon inflammation in colitis mice. Targeted metabolomics and 16S rRNA-based microbiota analysis revealed that TPS effectively alleviated dysbiosis of the gut microbiota, particularly by increasing the abundance of probiotics associated with tryptophan metabolism, such as Lactobacillus and Clostridia-UCG-014. The study also found that TPS exerted its gut barrier functions through the activation of the aryl hydrocarbon receptor (AhR), which upregulated epithelial tight junction proteins (39).

Intestinal alkaline phosphatase (IAP) has also been investigated for its role in relieving colitis. In a study involving obese mice subjected to forced exercise, IAP treatment significantly reduced disease activity, oxidative stress markers, and pro-inflammatory cytokines. IAP treatment also enhanced the expression of barrier proteins in the colonic mucosa and modified the gut microbiota composition, promoting the proliferation of beneficial Verrucomicrobiota while reducing the presence of pathogenic Clostridia and Odoribacter. These results indicate that IAP may serve as a promising therapeutic strategy for UC (40).

Desmethylbellidifolin, a xanthone isolated from *Gentianella acuta*, has shown anti-UC activity in DSS-induced colitis in mice. The treatment alleviated colon shortening, body weight loss, and lowered the disease activity index. It also ameliorated colonic inflammation by suppressing the expression of interleukin-6 and tumor necrosis factor-*α*, strengthened the intestinal epithelial barrier, and modulated gut dysbiosis (41).

Polyphenols extracted from Fu brick tea (FBTP) have demonstrated preventive effects on experimental colitis. FBTP supplementation mitigated colitis symptoms, immune cell infiltration, and the secretion of pro-inflammatory cytokine in DSS-induced colitis mice. The treatment modulated gut microbiota and facilitated the microbial conversion of tryptophan into indole-3-acetic acid (I3A), resulting in AhR-mediated protection against colitis. This protection was achieved through the upregulation of interleukin-22 (IL-22) and the expression of tight junction proteins (42).

5-Aminosalicylic acid (5-ASA) is widely used in clinical practice for UC treatment. A study found that 5-ASA ameliorated colitis symptoms in DSS-treated mice by reshaping the disordered gut microbiota community structure. The treatment promoted the abundance of Bifidobacterium, Lachnoclostridium, and Anaerotruncus, and improved the abnormal metabolism of bile acids by regulating the Farnesoid X receptor (FXR) and Takeda G-protein-coupled receptor 5 (TGR5) signaling pathways (43). Luteolin, a natural compound, has been documented to reduce inflammation and modulate gut microbiota in UC. In DSS-induced colitis rats, luteolin treatment significantly reduced colonic damage and inflammation altered the diversity and composition of gut microbiota, and increased the abundance of beneficial genera such as Lactobacillus and Bacteroides (44).

In conclusion, the modulation of gut microbiota presents a viable therapeutic approach for UC. Various natural compounds and treatments have shown efficacy in altering gut microbiota composition, reducing inflammation, and improving gut barrier function, thereby alleviating UC symptoms. Further research is needed to fully understand the mechanisms and optimize these treatments for clinical use.

### 1.5 The impact of antibiotics combined with biological agents on the treatment of ulcerative colitis

The combination of antibiotics and biological agents has been explored as a therapeutic strategy for UC, particularly in cases where conventional treatments fail. Antibiotics are primarily used to manage infections and modulate the gut microbiota, which plays a crucial role in the pathogenesis of UC. Biological agents, on the other hand, target specific components of the immune system to reduce inflammation and induce remission.

One of the key studies in this area evaluated the efficacy of combining antibiotics with biological agents in treating UC. The study found that the use of antibiotics, such as ciprofloxacin and metronidazole, in conjunction with anti-TNF agents like infliximab, resulted in improved clinical outcomes compared to the use of biological agents alone (45). This combination therapy was particularly effective in patients with refractory UC, where standard treatments had previously failed.

In another study, the Spanish Working Group on Crohn’s Disease and Ulcerative Colitis (GETECCU) recommended the use of antibiotics like Ciprofloxacin or rifaximina as a first-line treatment for acute pouchitis, a frequent complication in UC patients who have undergone ileal pouch-anal anastomosis (IPAA) surgery. The study also suggested that chronic pouchitis might benefit from a combination of antibiotics and biological agents, particularly in cases where patients are refractory to conventional treatments (46). Despite these promising findings, the combination of antibiotics and biological agents is not without risks. For instance, a case report highlighted the challenges of managing a patient with acute severe UC and concurrent SARS-CoV-2 infection. The patient was treated with infliximab and subsequently developed an infection with enterohemorrhagic *Escherichia coli*, necessitating antibiotic treatment (47). This case underscores the need for careful monitoring and individualized treatment plans when using combination therapies.

The safety profile of biological agents, such as vedolizumab, has also been a subject of investigation. A study comparing the infectious risks of vedolizumab with other biological agents found that patients treated with vedolizumab had a higher incidence of gut infections, likely due to its gut-selective mechanism of action (48). This finding suggests that while vedolizumab is effective, its use in combination with antibiotics should be carefully considered to mitigate the risk of infections.

In conclusion, the combination of antibiotics and biological agents offers a promising therapeutic approach for managing UC, particularly in refractory cases. However, the potential risks associated with this combination necessitate careful patient selection and monitoring. Future research should focus on optimizing treatment protocols to maximize efficacy while minimizing adverse effects.

## 2 Limits of the current study

There are some limits of the current study. Although the intricate relationship between the gut microbiota and UC stresses the critical role of microbial communities in the pathogenesis and treatment of this chronic inflammatory condition, the evidence presented in the reviewed studies highlights several key points that merit further discussion and exploration. Firstly, the intestinal microbiota’s composition and diversity are significantly altered in UC patients, suggesting a direct link between microbial imbalance and disease onset. The dysbiosis observed in these patients not only exacerbates intestinal inflammation but also disrupts the mucosal barrier, leading to increased disease severity. This underlines the importance of maintaining a balanced gut microbiome for intestinal health and raises the potential of microbiota-targeted therapies in UC management. Secondly, the role of extra-intestinal microbiota in UC pathogenesis remains a burgeoning field of study. While preliminary data indicate that microbial communities outside the gut may influence systemic inflammation and immune responses, more comprehensive research is needed to elucidate these mechanisms and their clinical implications. This area holds promise for identifying novel biomarkers and therapeutic targets for UC. The impact of gastroenterological microbiota on the efficacy of biologic therapies for UC is another critical aspect explored in this review. The interplay between microbial composition and patient response to biologics such as anti-TNF agents and integrin inhibitors suggests that microbiota profiling could become a valuable tool in personalizing UC treatment. By identifying microbial signatures associated with therapeutic success or failure, clinicians can tailor interventions to improve outcomes and reduce adverse effects. Moreover, the modulation of gastrointestinal microbiota through dietary interventions, probiotics, prebiotics, and FMT has demonstrated potential in alleviating UC symptoms and inducing remission. These strategies are designed to restore microbial equilibrium and promote mucosal healing, offering a complementary approach to conventional therapies. However, standardized protocols and long-term safety data are necessary to fully integrate these modalities into clinical practice. Finally, the combination of antibiotics with biologic agents presents a novel therapeutic avenue for UC. While antibiotics can reduce pathogenic bacteria and modulate the immune response, their use must be judicious to avoid resistance and further dysbiosis. The synergistic effects of antibiotics and biologics could potentially enhance treatment efficacy, but this approach requires careful consideration of patient-specific factors and microbial dynamics.

## 3 Conclusion

The complex interplay between gut microbiota or oral microbiota and UC offers both challenges and opportunities for further investigation. Future research should aim to elucidate the specific mechanisms underlying microbial influence on UC, developing robust microbiota-based diagnostics, and optimizing therapeutic strategies that incorporate microbial modulation. By balancing the diverse perspectives and findings in this field, we can advance towards more effective and personalized treatments for UC, ultimately improving patient outcomes and quality of life.
